# Supplementary figures and images for: Comprehensive analyses of genomes, transcriptomes and metabolites of neem tree
Source: PeerJ. 2015 Aug 6;3:e1066. doi: 10.7717/peerj.1066 (PMC4540028; doi:10.7717/peerj.1066)

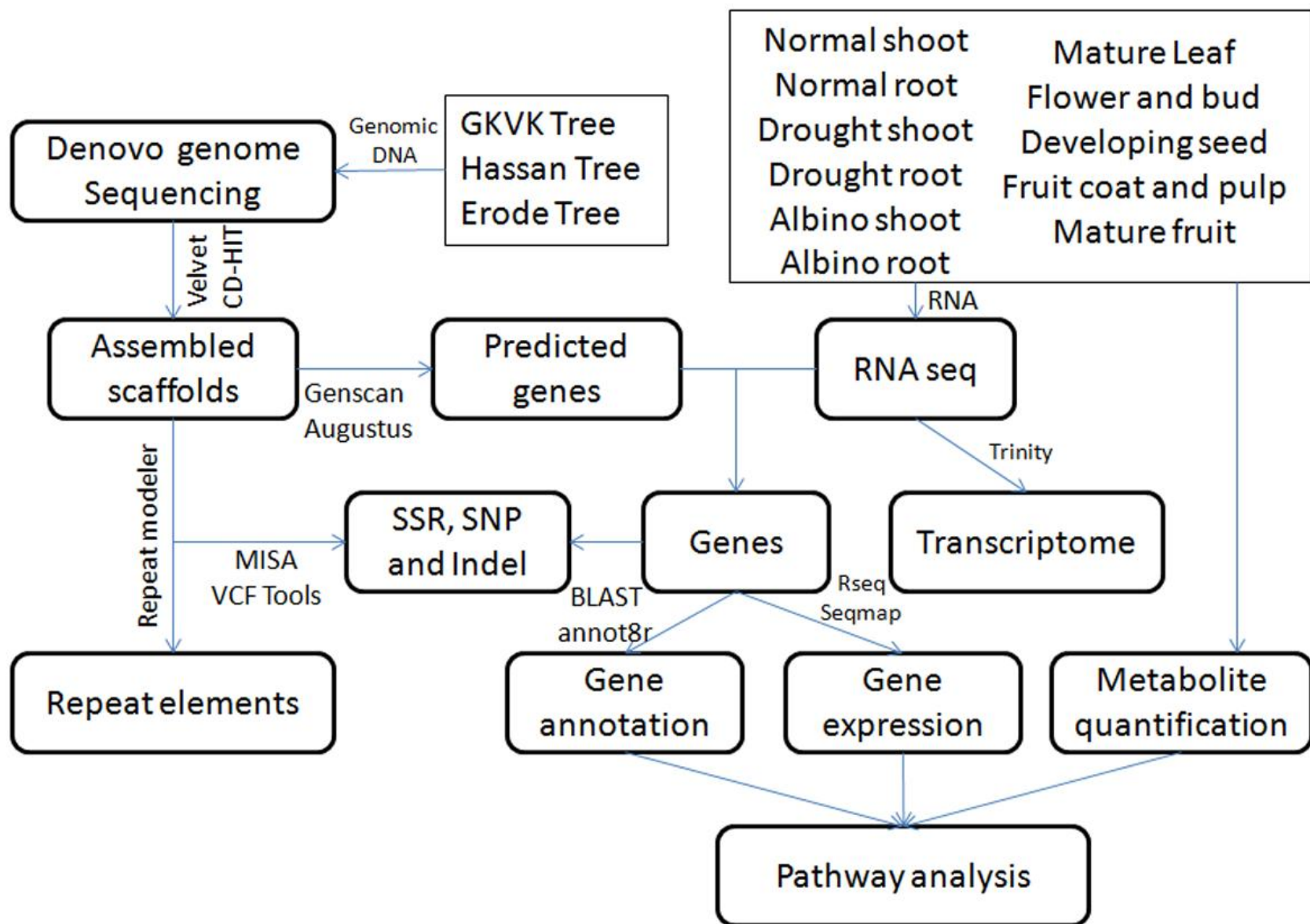

Supplement: Figure S1 [file peerj-03-1066-s021.pdf]

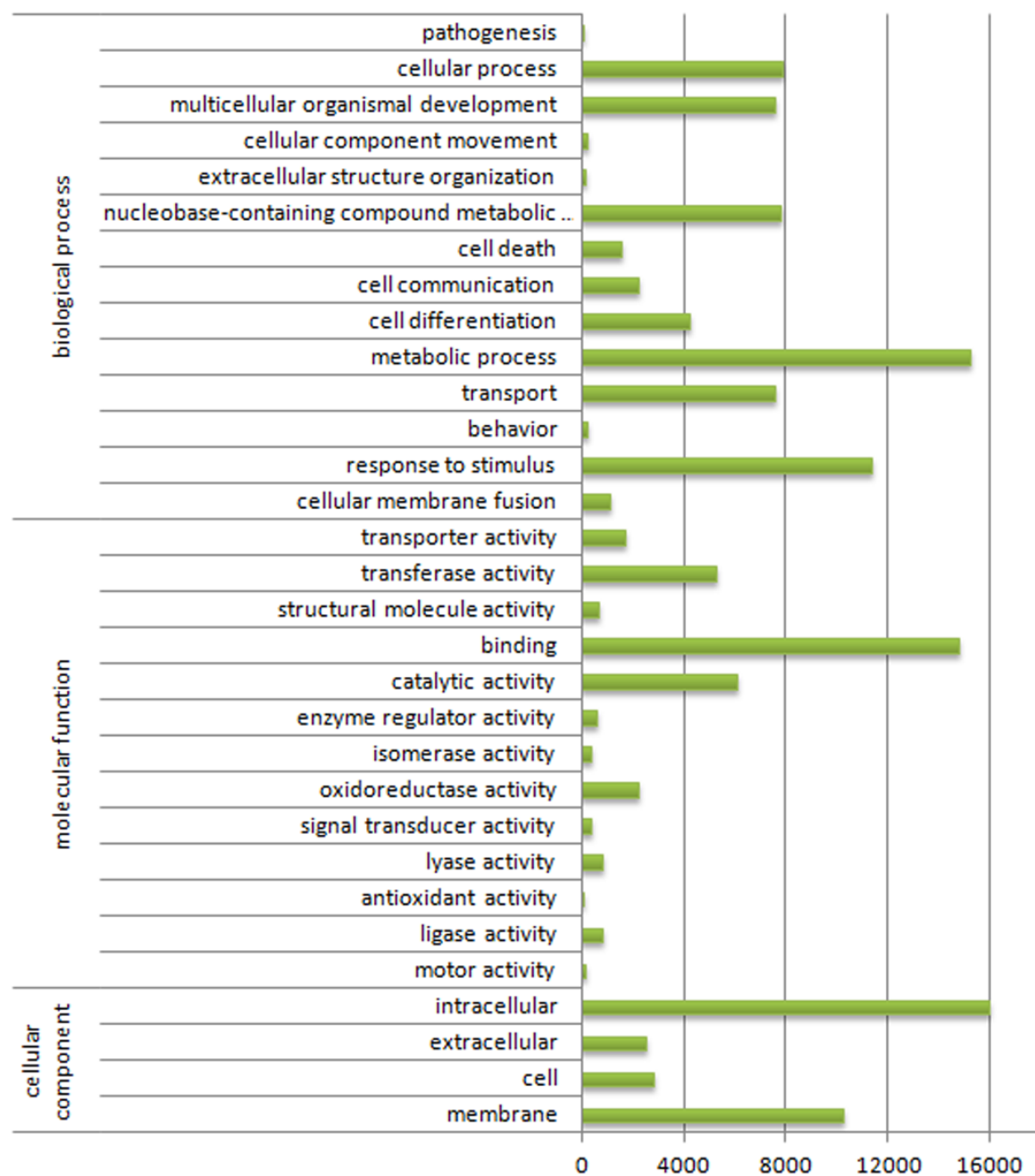

Supplement: Figure S2 [file peerj-03-1066-s022.pdf]
